# Supplementary material for: Constraining the equation of state in neutron-star cores via the long-ringdown signal
Source: Nat Commun. 2025 Feb 3;16:1320. doi: 10.1038/s41467-025-56500-x (PMC11790964; doi:10.1038/s41467-025-56500-x)
Supplement: Supplementary file 1 — Supplementary Information [file 41467_2025_56500_MOESM1_ESM.pdf]

Supplementary Information: Constraining the equation of state in neutron-star cores via the long ringdown signal

Christian Ecker[1*], Tyler Gorda[1,2,3*], Aleksi Kurkela[4*] and Luciano Rezzolla[1,5,6*]

[1]Institut für Theoretische Physik, Goethe Universität, 60438, Frankfurt am Main, Germany.
[2]ExtreMe Matter Institute EMMI, GSI Helmholtzzentrum für Schwerionenforschung GmbH, 64291, Darmstadt, Germany.
[3]Department of Physics, Technische Universität Darmstadt, 64289 Darmstadt, Germany.
[4]Faculty of Science and Technology, University of Stavanger, Stavanger, 4036, Stavanger, Norway.
[5]Frankfurt Institute for Advanced Studies, 60438, Frankfurt, Germany.
[6]School of Mathematics Trinity College, Dublin 2, Ireland.
[7]Corresponding author.

*Corresponding author(s). E-mail(s): ecker@itp.uni-frankfurt.de; gorda@itp.uni-frankfurt.de; aleksi.kurkela@uis.no; rezzolla@itp.uni-frankfurt.de;

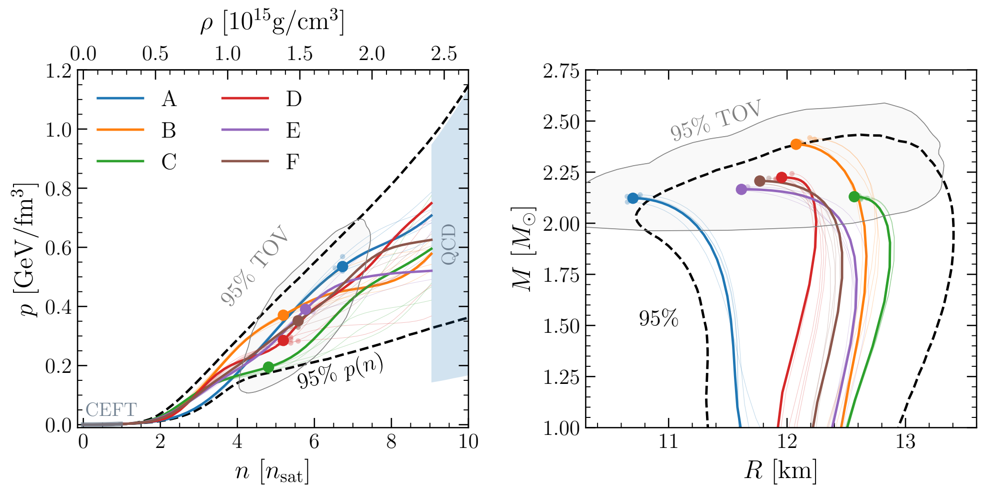

Supplementary Figure 1 Golden and nearby EOSs. Similar to Fig. 1 of the main text, with the golden EOSs shown in thick colored lines and the TOV points shown with large filled points. Additionally shown with thin lines are the five next-highest likelihood EOSs from the 30 closest EOSs to the points on the 68% contour. The TOV points for these nearby EOSs are also shown with small filled points. Source data for this figure are provided as a Source Data file.

Supplementary Note 1: Robustness of the Principal-component Analysis

Supplementary Figure 1 shows the golden EOSs selected by the principal-component analysis outlined in the Methods section as curves in the (p, n) and (M, R) planes (thick solid colored lines), as well as the five next-highest likelihood EOSs from the 30 nearby EOSs (thin solid colored lines). Note that at least for densities $n \leq n_{\rm TOV}$, the selection procedure is robust, with the nearby EOSs having a similar structure to the corresponding golden EOSs.

Supplementary Note 2: Details of the simulated binaries

EOS	$M_{\rm TOV}$ $[M_\odot]$	$C_{\rm TOV}$	$R_{1.4}$ [km]	$p_{\rm TOV}$ $[\rm GeV/fm^3]$	$n_{\rm TOV}$ $[n_{\rm sat}]$	q	$\tilde{\Lambda}$	f_2 [Hz]	$f_{\rm rd}$ [Hz]	$d\widehat{E}_{\rm GW}/d\widehat{J}_{\rm GW}$	$\sigma_{d\widehat{E}_{\rm GW}/d\widehat{J}_{\rm GW}}$
A	2.12	0.293	11.55	0.535	6.73	0.70	286	3380	3366	3.48	0.08
						0.85	301	3245	3344	3.38	0.04
						1.00	303	3205	3229	3.25	0.09
B	2.39	0.292	12.57	0.370	5.19	0.70	528	2600	2587	3.02	0.06
						0.85	538	2710	2601	2.91	0.08
						1.00	566	2725	2716	3.00	0.05
C	2.13	0.250	12.72	0.195	4.81	0.70	520	2495	2471	2.85	0.08
						0.85	577	2615	2695	2.85	0.12
						1.00	627	2635	2611	2.89	0.05
D	2.22	0.275	12.06	0.285	5.19	0.70	352	2680	2677	2.93	0.10
						0.85	358	2900	2759	2.98	0.08
						1.00	434	2850	2883	3.00	0.10
E	2.17	0.275	12.53	0.390	5.77	0.70	520	2665	2637	3.08	0.06
						0.85	540	2700	2687	2.95	0.08
						1.00	562	2756	2761	3.05	0.05
F	2.21	0.277	12.37	0.352	5.58	0.70	456	2680	2662	2.99	0.07
						0.85	494	2735	2625	2.94	0.09
						1.00	502	2815	2802	3.07	0.06
DD2	2.41	0.299	13.20	0.544	5.42	1.00	777	2590	2574	2.71	0.06
V-QCD	2.14	0.265	12.47	0.296	5.09	1.00	565	2860	2852	2.87	0.04

Supplementary Table 1 EOS, NS and BNS properties. For each EOS, we list the TOV-mass $M_{\rm TOV}$, the TOV compactness $C_{\rm TOV}$, the radii of a $1.4\ M_\odot$ NS $R_{1.4}$, the TOV pressure $p_{\rm TOV}$, the TOV number density $n_{\rm TOV}$, the binary tidal deformability $\tilde{\Lambda}$, the post-merger frequencies f_2, the long-ringdown frequency $f_{\rm rd}$ and the corresponding slope $d\widehat{E}_{\rm GW}/d\widehat{J}_{\rm GW}$ and standard deviation of the slope $\sigma_{d\widehat{E}_{\rm GW}/d\widehat{J}_{\rm GW}}$. Source data for this table are provided as a Source Data file.

Supplementary Table 1 provides a concise summary of the most salient properties of the binaries with the golden EOSs that have been simulated, together with the characteristic GW frequencies f_2 and $f_{\rm rd}$.

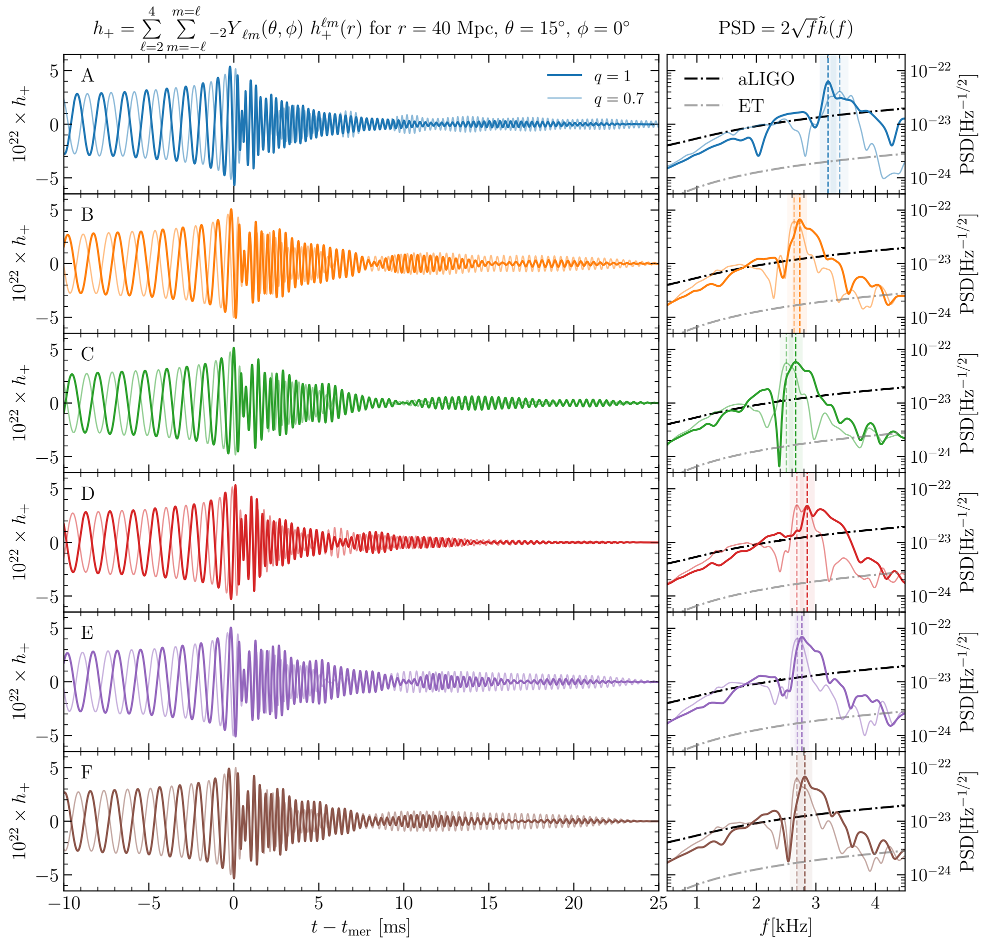

Supplementary Figure 2 Gravitational Waves GW strain (left) and post-merger power spectral density (right) at 40 Mpc detector distance and $\theta = 15°$ viewing angle for equal chirp mass $M_c = 1.18\, M_\odot$ and two different mass ratios $q = 1, 0.7$. In the right panel we mark with solid lines the dominant post-merger frequency f_2, where the shaded areas indicate a 8% relative error estimate. Source data for this figure are provided as a Source Data file.

Supplementary Note 3: Merger Simulations and GW Analysis

Supplementary Figure 2 summarises the GW output from a number of our simulations by reporting on the left column the GW strain and on the right column the corresponding PSD from the post-merger signal when compared with the estimated sensitivities of advanced LIGO (aLIGO) and the Einstein Telescope (ET). The data refers to BNS simulations for the EOSs A-F (top to bottom), all having the same chirp mass $\mathcal{M}_c = 1.18\, M_\odot$ and two different mass ratios $q = 1, 0.7$ (dark and light colors, respectively). Consistent with the expectations from the GW170817 event, the results shown assume a distance of $40\mathrm{Mpc}$ and a viewing angle of $\theta = 15°$.

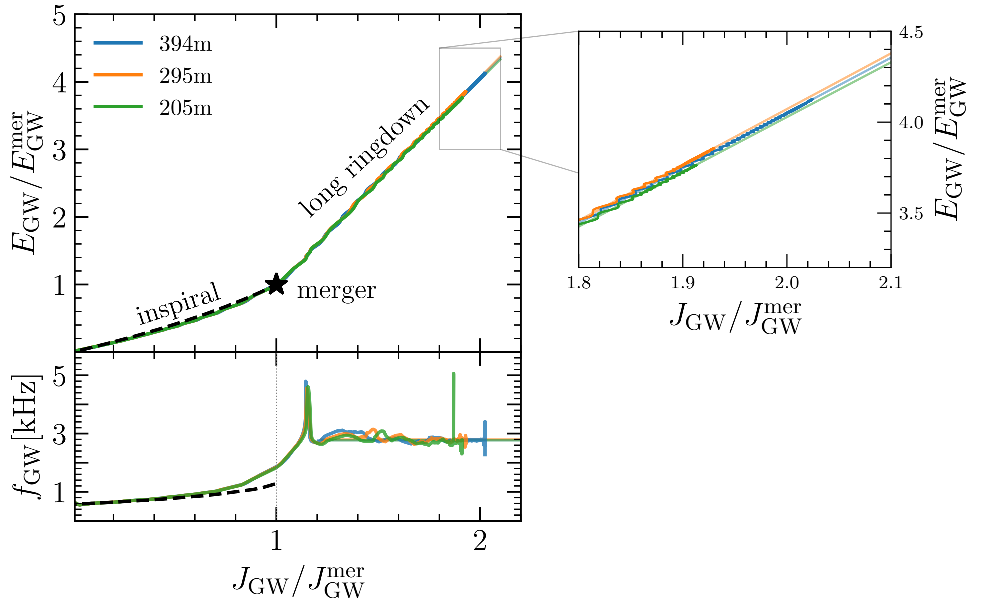

Supplementary Figure 3 Resolution dependence. GW energy as function of the angular momentum (top) and the corresponding GW frequency (bottom) for different values of the grid resolution. The examples shown are for equal-mass binaries and the EOS F. Source data for this figure are provided as a Source Data file.

Next, we demonstrate the robustness of our long-ringdown slope computation with respect to the grid resolution used in the numeric simulation. To this scope, we performed, in addition to our standard resolution ($295\,\mathrm{m}$), also simulations with higher ($205\,\mathrm{m}$) and lower ($394\,\mathrm{m}$) resolutions. The results of these simulations are summarized in Supplementary Figure 3, which highlights how the slope is essentially insensitive to the resolution and that even simulations with low resolutions result in slope values that are well within the uncertainty of the fit (± 0.1, see discussion below). More specifically, we measure slopes of $\{2.99 \pm 0.06, 3.06 \pm 0.07, 3.02 \pm 0.06\}$ for grid-resolutions of $\{205\,\mathrm{m},\ 295\,\mathrm{m},\ 394\,\mathrm{m}\}$, respectively. More importantly, the measure of the slope at different resolutions displays a much smaller variance than the equivalent measure of f_{rd} (see lower panel of Supplementary Figure 3). The somewhat surprising robustness of the long-ringdown slope with resolution can be simply explained by the fact that the post-merger waveform is dominated by the large-scale $\ell = 2, m = 2$ deformations of the merger remnant, which are only weakly influenced by the small-scale features within the remnant.

Finally, we comment on the accuracy of the slope extraction from the numerical simulations. This operation requires first to identify the optimal time-range for the linear least-squares fit of $E_{\mathrm{GW}}(J_{\mathrm{GW}}(t))$, whose starting and final times are determined by minimizing the variance of the linear fit. More specifically, we first compute the slope and its variance for a number of different starting times $t_{\mathrm{in}} - t_{\mathrm{mer}} \in [1 - 10]\,\mathrm{ms}$ using a fixed value for the final time $t_{\mathrm{fin}} - t_{\mathrm{mer}} = 15\,\mathrm{ms}$. In this way, we found that a starting time of $t_{\mathrm{in}} - t_{\mathrm{mer}} = 1\,\mathrm{ms}$ results in an approximate variance of ± 0.1 for the slope. Larger values for the starting time, e.g., $5\,\mathrm{ms}$ or $10\,\mathrm{ms}$, result in significantly larger variances of ± 0.7 and

± 3.1, respectively. Next, we compute the slope and its variance for various values of the final time $t_{\rm fin} - t_{\rm mer} \in [2-25]\,{\rm ms}$ while keeping the starting time fixed at $t_{\rm in} - t_{\rm mer} = 1\,{\rm ms}$. In this way, we found that the variance saturates at $t_{\rm fin} - t_{\rm mer} \approx 15\,{\rm ms}$ to values similar to those obtained by varying the starting time. Increasing the final time of the fit does not lead to further improvement of the fit quality and this is because it becomes increasingly difficult to accurately compute the small changes in $E_{\rm GW}$ and $J_{\rm GW}$ at times beyond $t - t_{\rm mer} \gtrsim 15\,{\rm ms}$, where the amplitude of the GW signal becomes very small. We apply an analogous procedure to determine $f_{\rm rd}$.

	β_0	β_1	β_2	β_3	β_4	β_5	β_6
β_0	4.16						
β_1	−6.40	10.59					
β_2	2.57	−6.32	11.27				
β_3	−3.86	6.24	−4.52	4.48			
β_4	6.15	−10.68	9.15	−7.22	12.46		
β_5	−4.39	8.85	−9.86	5.29	−10.48	10.97	
β_5	1.76	−2.08	−3.25	−0.07	−0.08	0.41	3.83

Supplementary Table 2 Covariance matrix of the bilinear model The covariance matrix $\mathrm{cov}(\boldsymbol{\beta})$ of the fitted bilinear model used in this work in Eq. (1) of the main text. The covariance matrix is symmetric. Here, the model has been fitted using a least-squares fit to the inputs where $p_{\rm TOV}$ is in units of $\mathrm{GeV/fm^3}$ and $n_{\rm TOV}$ is in fm^{-3}. Source data for this table are provided as a Source Data file.

Supplementary Note 4: Performing the mock measurement

We perform a mock joint measurement of f_2 and the slope $d\widehat{E}_{\rm GW}/d\widehat{J}_{\rm GW}$ in the following manner. We assume a measurement whose uncertainty we model with a multivariate Gaussian distribution of f_2 and $d\widehat{E}_{\rm GW}/d\widehat{J}_{\rm GW}$ for simplicity, as well as a uniform measurement of $q \in [0.7, 1.0]$. Let us denote the joint likelihood from the measurement $P_{\rm meas}(\mathrm{data}|f_2, d\widehat{E}_{\rm GW}/d\widehat{J}_{\rm GW}, q)$. First, we fit a two-component model to the f_2 and $d\widehat{E}_{\rm GW}/d\widehat{J}_{\rm GW}$ data, the posterior of which we denote by $P_{\rm mod}(d\widehat{E}_{\rm GW}/d\widehat{J}_{\rm GW}|\mathrm{EOS}, q)$ respectively. This two-component model is just the product of two models of the form given by Eq. (1) in the main text for f_2 and $d\widehat{E}_{\rm GW}/d\widehat{J}_{\rm GW}$. Next, we compute the likelihood that each EOS is consistent with the mock measurement by evaluating

$$P(\mathrm{data}|\mathrm{EOS}, q) = \int df_2 \, d\Big(d\widehat{E}_{\rm GW}/d\widehat{J}_{\rm GW}\Big) \times P_{\rm meas}(\mathrm{data}|f_2, d\widehat{E}_{\rm GW}/d\widehat{J}_{\rm GW}, q) \times P_{\rm mod}(d\widehat{E}_{\rm GW}/d\widehat{J}_{\rm GW}, f_2|\mathrm{EOS}, q)\,, \tag{1}$$

by Monte-Carlo sampling of the measurement distribution, which we then use in Bayes's theorem to generate the posteriors in Fig. 4 of the main text, using a flat prior on $q \in [0.7, 1.0]$. The likelihoods when using only one of the two bilinear models is defined similarly.

The bilinear model of Eq.(1) in the main text reproduces well long-ringdown slopes, where the distribution of model parameters $\boldsymbol{\beta} := (\beta_0, \ldots, \beta_6)$ are given by a multivariate Gaussian distribution with a mean $\bar{\boldsymbol{\beta}} = (1.78, 0.72, -1.44, 1.90, -1.74, -1.14, 3.61)$ and a covariance matrix $\mathrm{cov}(\boldsymbol{\beta})$ reported in Supplementary Table 2, with $p_{\rm TOV}$ and $n_{\rm TOV}$ expressed in units of $\mathrm{GeV/fm^3}$ and fm^{-3}, respectively.

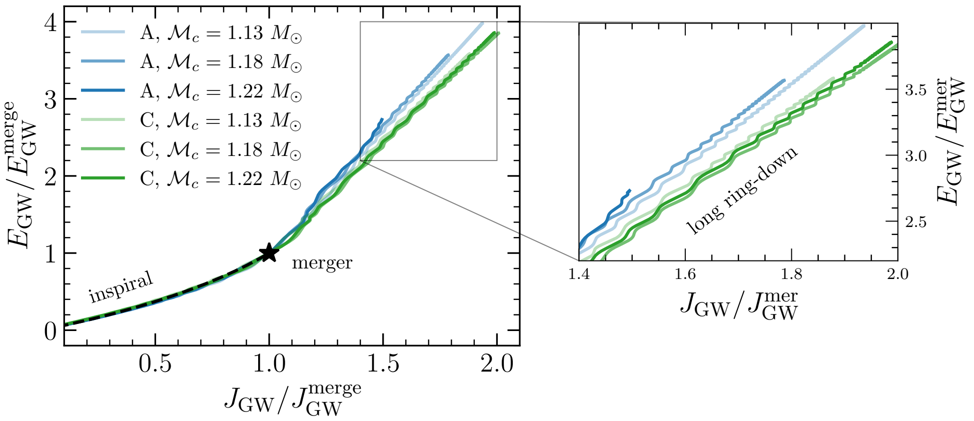

Supplementary Figure 4 Impact of chirp mass relation between normalized GW energy and angular momentum for model A (blue) and C (green) for three different values of the chirp mass $\mathcal{M}_{\rm chirp} = 1.13, 1.18, 1.22\, M_\odot$ (light to dark colors) and fixed mass ratio $q = 1$. Source data for this figure are provided as a Source Data file.

Supplementary Note 5: On the robustness of the correlation

In order to identify potential degeneracies in the long-ringdown slope between the chirp mass and the EOS properties we simulate the equal-mass ($q = 1$) binaries with EOS A and C with three different values for the chirp mass, namely, $\mathcal{M}_{\rm chirp} = 1.13,\ 1.18,\ 1.22\, M_\odot$. Since the chirp mass represents one of the best-measured quantities in BNS mergers with a few-percent error, what we are assessing in this way is the dependence for a given EOS of the long-ringdown slope on $\mathcal{M}_{\rm chirp}$. Stated differently, we can assess how different long-ringdown slopes cluster when exploring the possible ranges in the chirp mass.

The result of this test are displayed in Supplementary Figure 4, which is similar to Fig. 2 of the main text, but where we show the slope for model A in green and for model C in blue, while light to dark colors indicate small to large values of $\mathcal{M}_{\rm chirp}$, respectively. Evidently, the variations in the chirp mass lead to significantly smaller differences in the long-ringdown slope than those introduced by the EOSs. Hence, Supplementary Figure 4 highlights that the EOS represents the dominant contribution to the long-ringdown slope and that the chirp mass plays only a sub-dominant role. This is natural to expect since the long-ringdown slope is essentially set by the equilibrium of the HMNS, which, in turn, is predominantly determined by the EOS.

Next, we show in Supplementar Figure 5 results analogous to Fig. 2 of the main text, but for mass ratios $q = 0.85$ and $q = 0.7$, which complement the information shown in Fig. 3 in the main text. Note that also the unequal-mass binaries show a clear linear correlation in the radiated energy and angular momentum during the long ringdown and that different mass ratios lead to slightly different slopes. We should also remark that over the timescale considered here, the $\ell = 2, m = 2$ mode is still the dominant one and the contributions from the $\ell = 2, m = 1$ mode are at least two orders of magnitude smaller. However, it is possible at later times that the $\ell = 2, m = 1$ mode will dominate (see, e.g., [**?**]) both for highly asymmetric binaries (for which the $m = 1$ deformation is quite large right after merger), but also for equal-mass binaries (for which the $m = 1$ asymmetry is initially small but grows

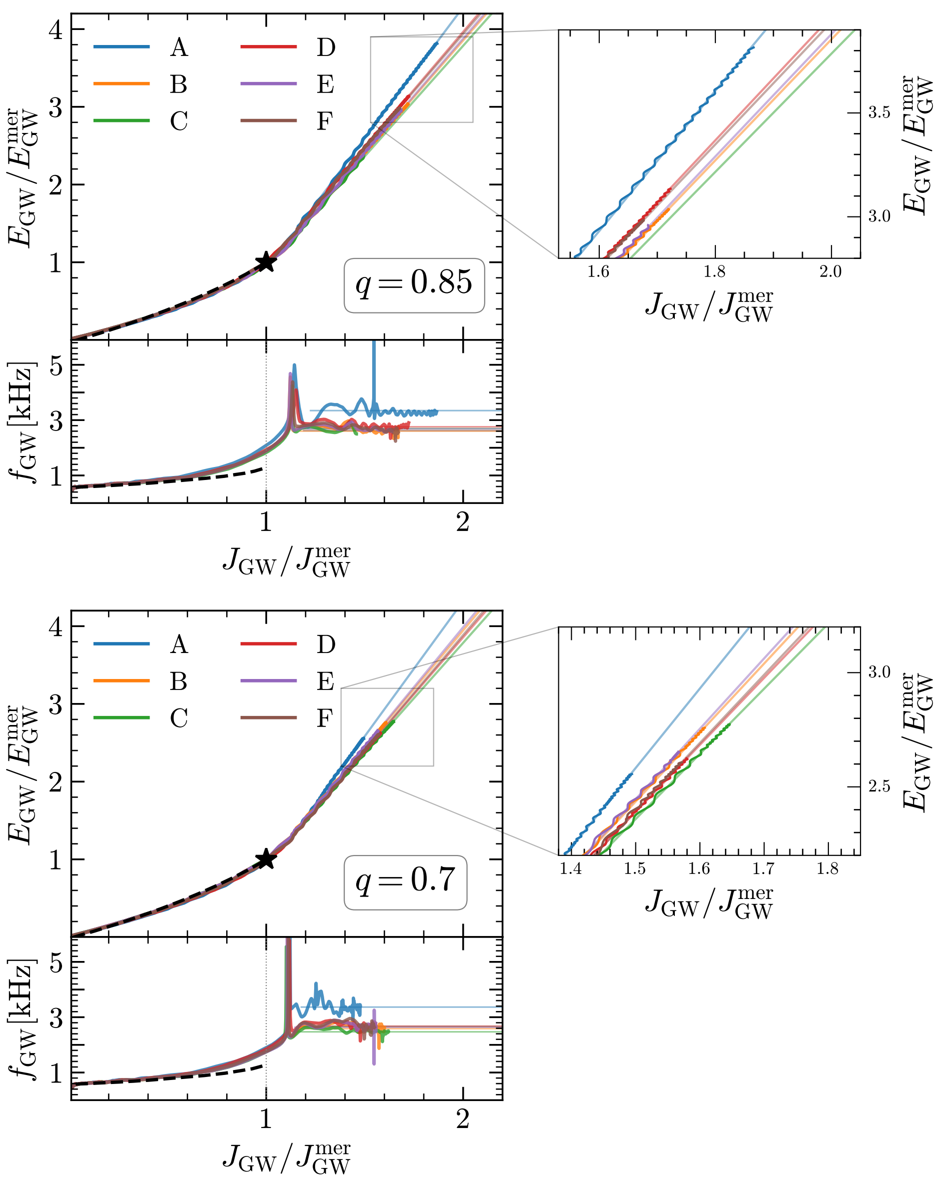

Supplementary Figure 5 Unequal-mass binaries Evolution of the normalized GW energy and GW frequency as a function of the normalized radiated angular momentum for the case of asymmetric binaries with mass ratio $q = 0.85$ (left) and $q = 0.7$ (right). Source data for this figure are provided as a Source Data file.

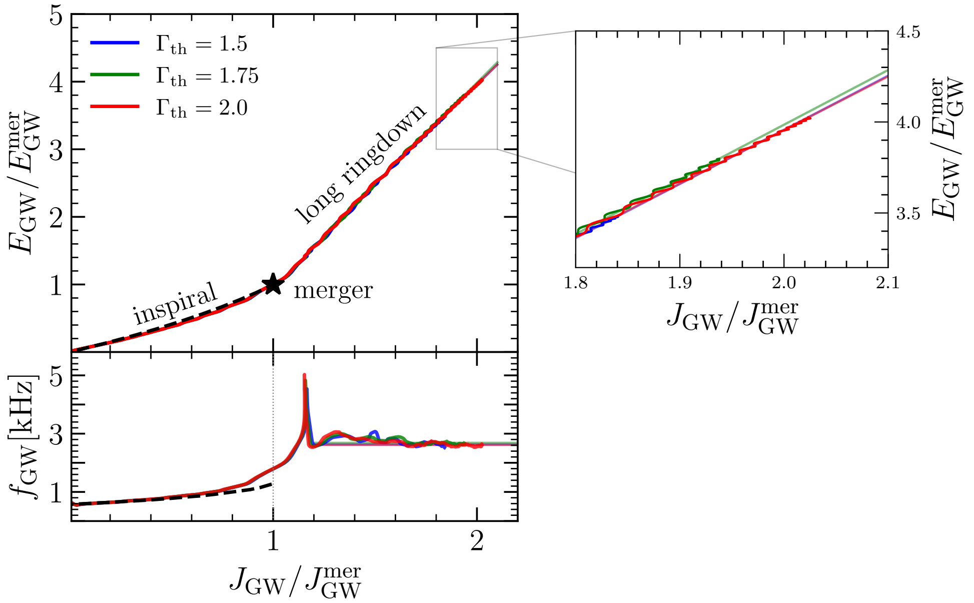

Supplementary Figure 6 Impact of Γ_{th} Same as Supplementary Figure 4 but for model B with three different values of $\Gamma_{th} = 1.5, 1.75, 2.0$ and fixed binary parameters $\mathcal{M}_c = 1.18$ and $q = 1$. Source data for this figure are provided as a Source Data file.

steadily). Also for this $\ell = 2, m = 1$ GW mode, the radiated energy and angular momentum will remain linearly related, albeit with a different (smaller) slope.

Another potential source of uncertainty in our results may have come from the thermal part of the EOS, which is admittedly simplified but qualitatively correct. In order to assess the impact of the thermal contributions, we study a reference EOS B with three different values that span the possible range expected for the adiabatic index, i.e., $\Gamma_{th} = 1.5, 1.75, 2.0$. The results of this analysis are reported in Supplementary Figure 6, which shows how larger values of Γ_{th} typically lead to higher thermal-pressure contributions, which help support the $\ell = 2 = m$ deformation of the merger remnant, and thus result in a more efficient GW emission in the post-merger phase. At the same time, the long-ringdown slope is essentially unaffected by the choice of Γ_{th} as can be seen from the tight overlap of the corresponding curves. As a result, we can conclude that our choice of a fiducial value of $\Gamma_{th} = 1.75$ does not introduce any bias on the reported long-ringdown slopes.

Finally, in Supplementary Figure 7 we show results for two EOSs with a tabulated temperature dependence, namely the Hempel-Schaffner DD2 (HS-DD2) EOS [**?**] and the intermediate variant of the holographic Veneziano QCD (V-QCD) EOS [**?**]. While the HS-DD2 EOS models purely hadronic matter, the V-QCD EOS features a strong first-order phase transition from hadronic to quark matter that induces the HMNS to collapse to a black hole in this simulation. As shown in [**?**], the strong phase transition of the V-QCD EOS does not allow stable quark-matter cores inside isolated stars, but a significant amount of quark matter can be formed during the metastable post-merger phase. Importantly, neither the presence of

quark matter, nor the microscopic prescription for the temperature dependence in these EOSs alter the basic features of the long ringdown.

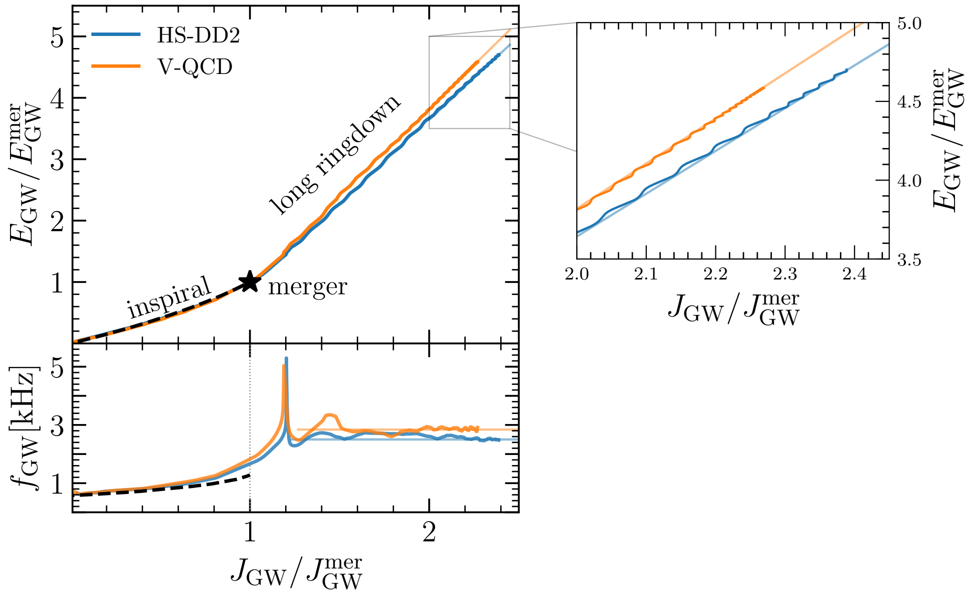

Supplementary Figure 7 Temperature dependent EOSs Same as Supplementary Figure 4, but for two models with tabulated temperature dependence, namely the HS-DD2 EOS and the intermediate variant of the holographic V-QCD EOS. Both simulations assume $q = 1$ and $\mathcal{M}_{\rm chirp} = 1.18\ M_\odot$. Source data for this figure are provided as a Source Data file.
