# Supplementary material for: Constraining the equation of state in neutron-star cores via the long-ringdown signal
Source: Nat Commun. 2025 Feb 3;16:1320. doi: 10.1038/s41467-025-56500-x (PMC11790964; doi:10.1038/s41467-025-56500-x)
Supplement: Supplementary file 3 — Source Data [file 41467_2025_56500_MOESM3_ESM.zip › Source_data/fig02_supplementary/GWq0p7and1.pdf]

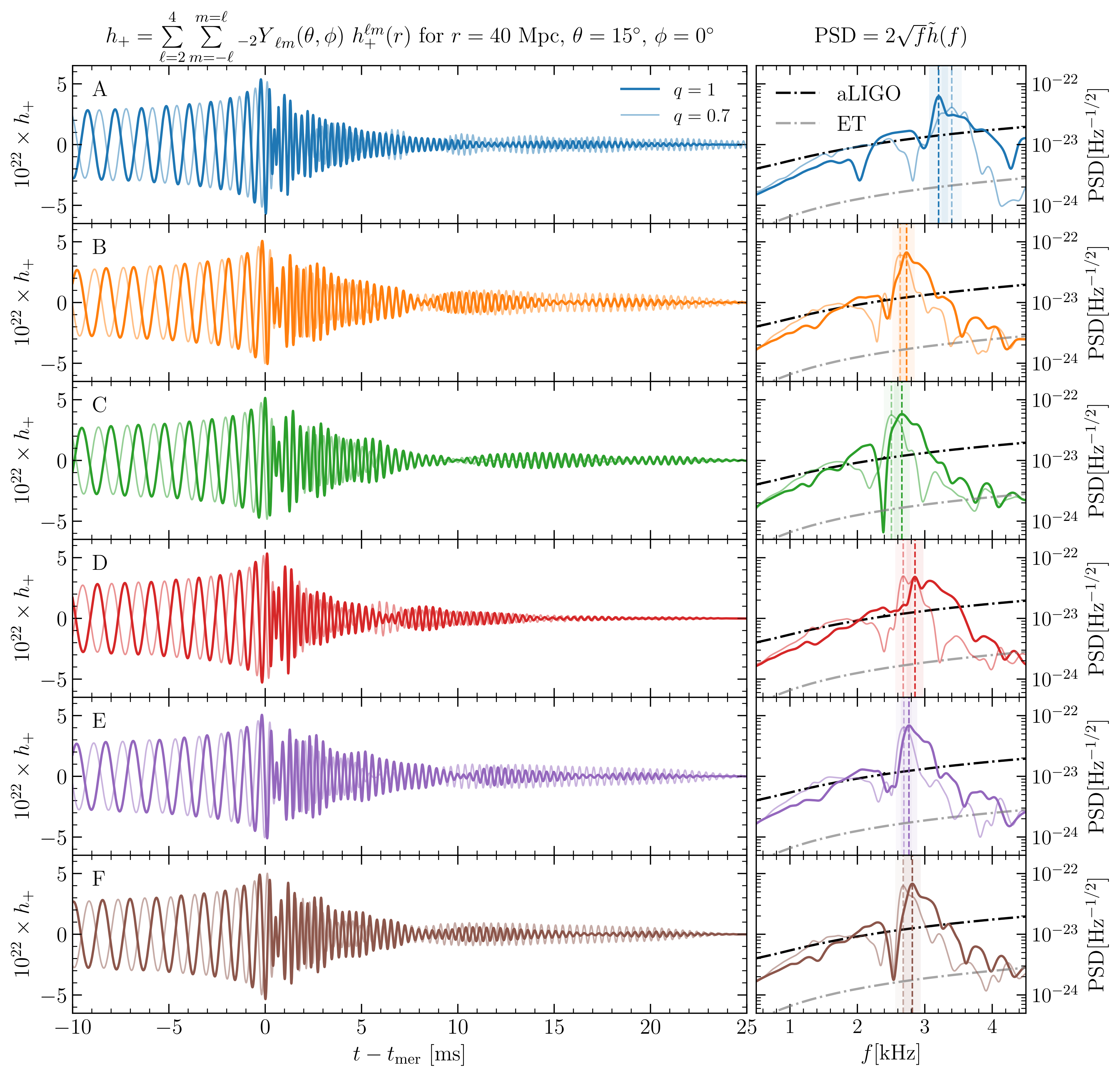
$h_+ = \sum_{\ell=2}^{4} \sum_{m=-\ell}^{m=\ell} {}_{-2}Y_{\ell m}(\theta,\phi)\, h_+^{\ell m}(r)$ for $r = 40$ Mpc, $\theta = 15°$, $\phi = 0°$
PSD $= 2\sqrt{f}\tilde{h}(f)$
A
B
C
D
E
F
$q = 1$
$q = 0.7$
aLIGO
ET
$10^{22} \times h_+$
$\mathrm{PSD}[\mathrm{Hz}^{-1/2}]$
$t - t_{\mathrm{mer}}$ [ms]
f[kHz]
