# Supplementary figures and images for: Constraining the equation of state in neutron-star cores via the long-ringdown signal
Source: Nat Commun. 2025 Feb 3;16:1320. doi: 10.1038/s41467-025-56500-x (PMC11790964; doi:10.1038/s41467-025-56500-x)

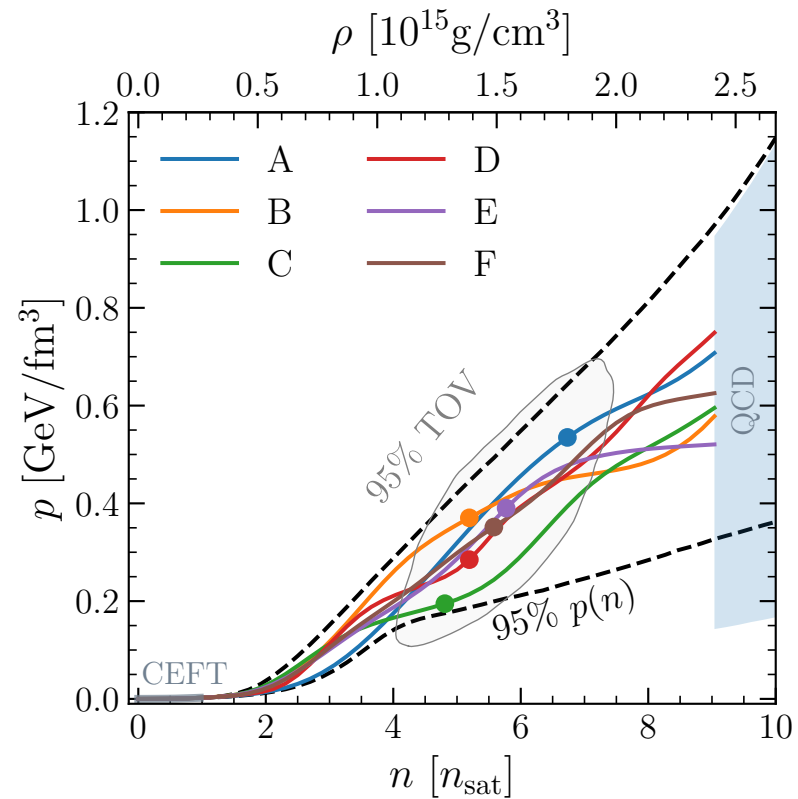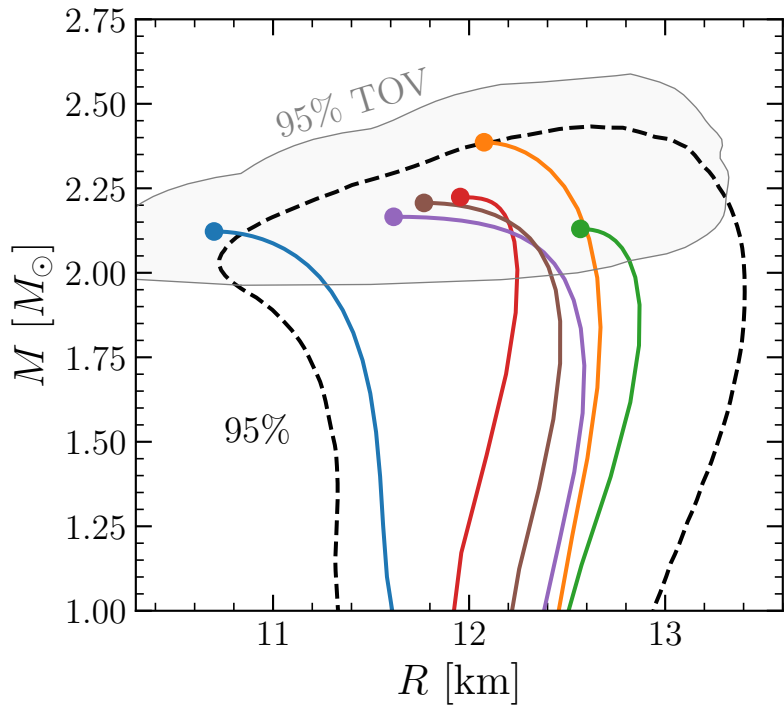

Supplement: Supplementary file 3 — Source Data [file 41467_2025_56500_MOESM3_ESM.zip › Source_data/fig01_main/fig01.pdf]

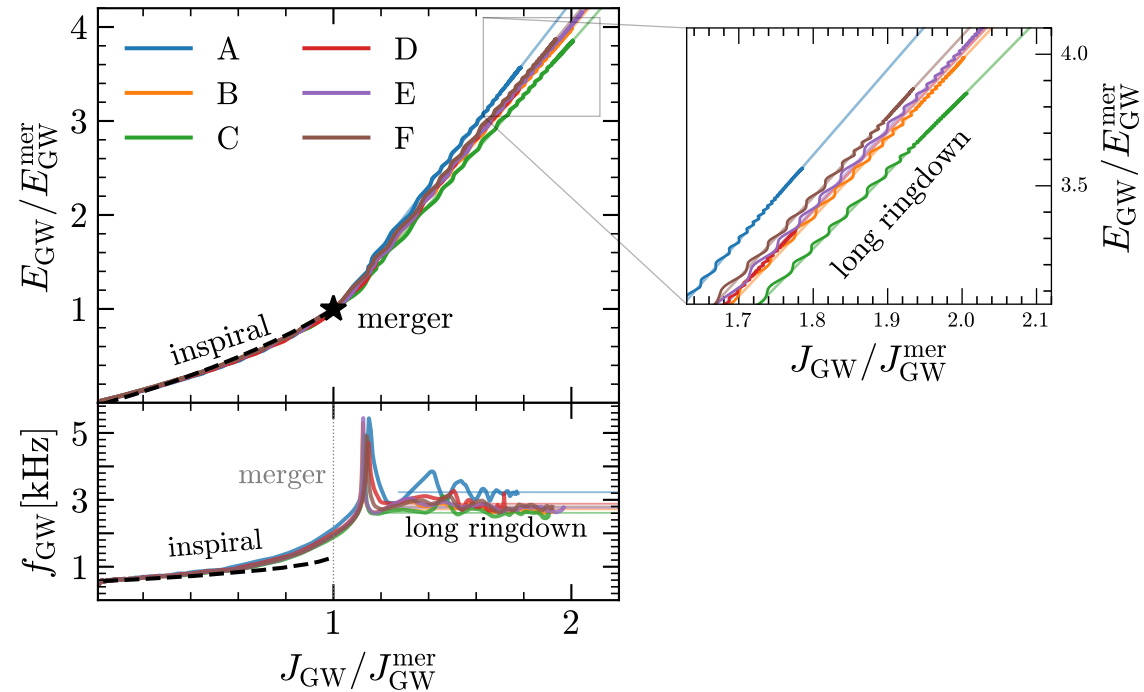

Supplement: Supplementary file 3 — Source Data [file 41467_2025_56500_MOESM3_ESM.zip › Source_data/fig02_main/fig02.pdf]

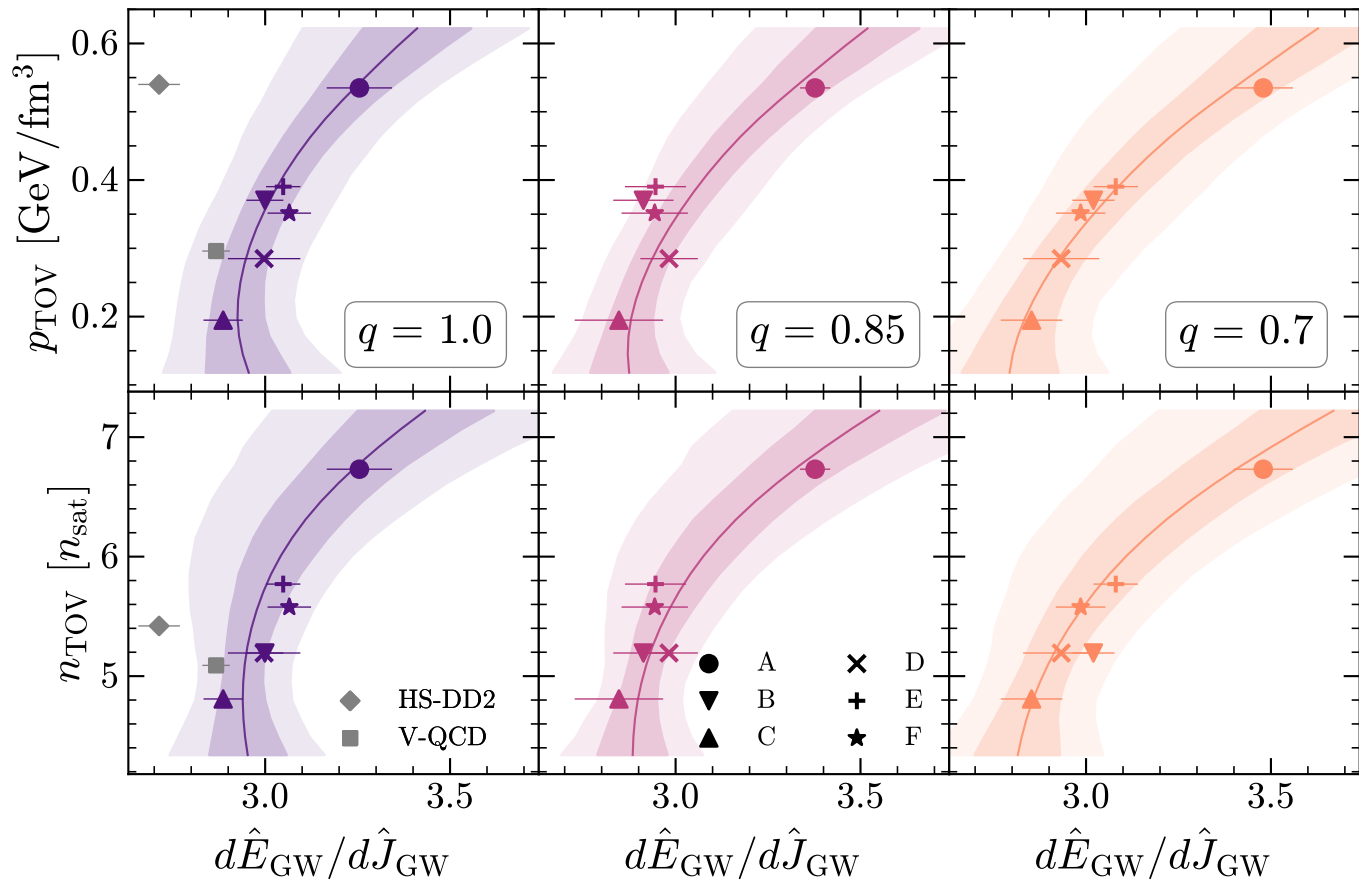

Supplement: Supplementary file 3 — Source Data [file 41467_2025_56500_MOESM3_ESM.zip › Source_data/fig03_main/fig03.pdf]

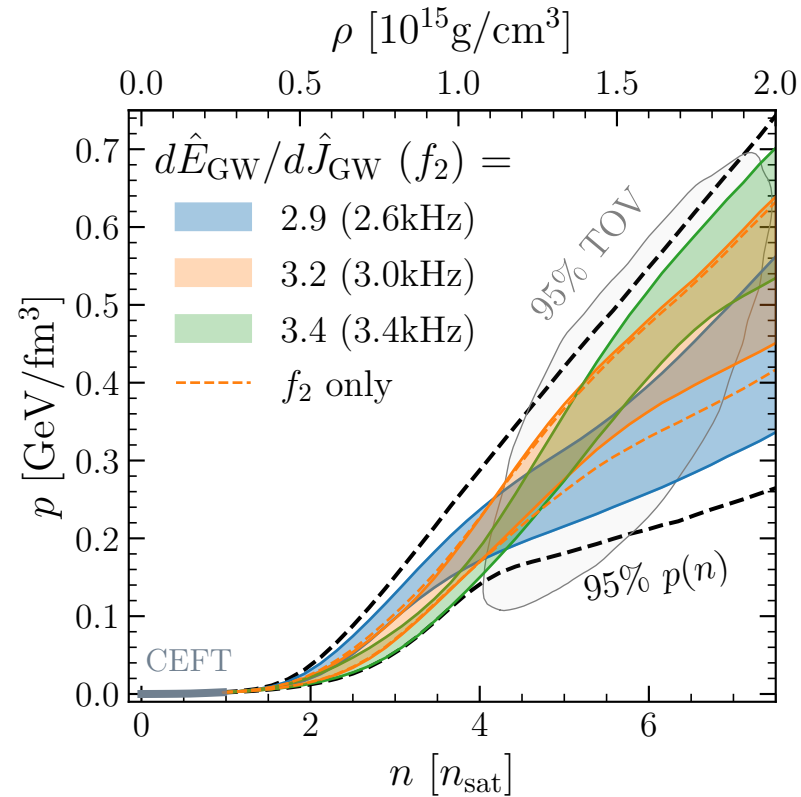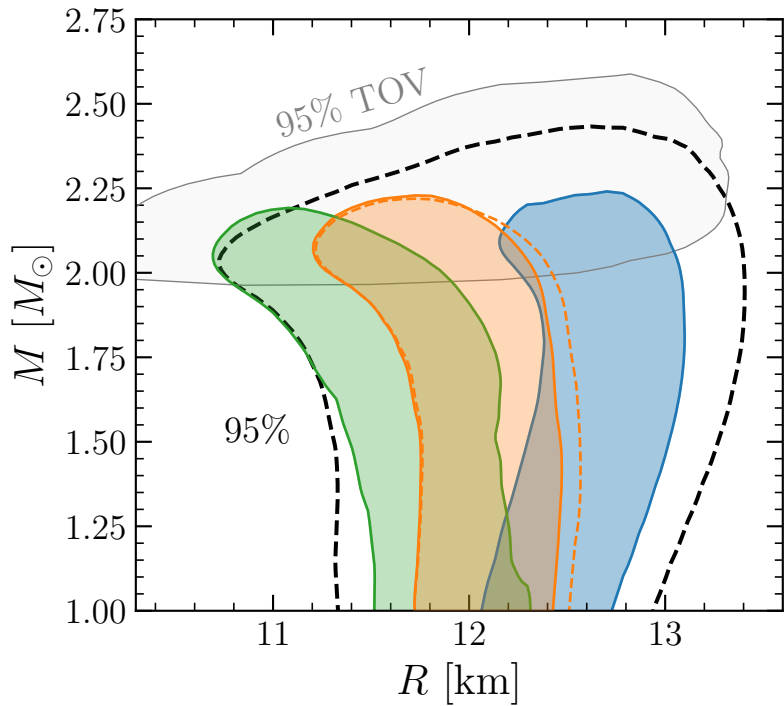

Supplement: Supplementary file 3 — Source Data [file 41467_2025_56500_MOESM3_ESM.zip › Source_data/fig04_main/fig04.pdf]

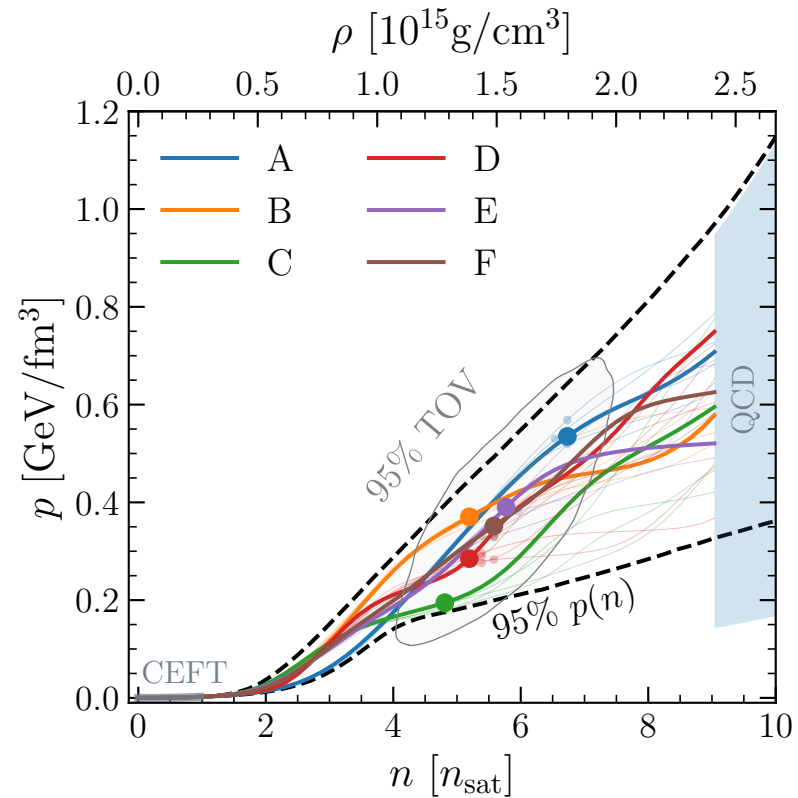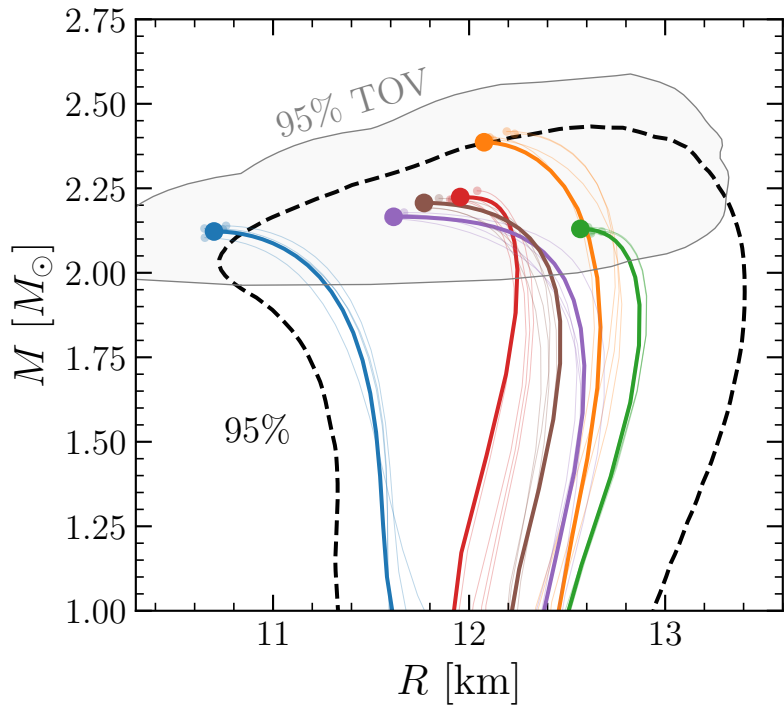

Supplement: Supplementary file 3 — Source Data [file 41467_2025_56500_MOESM3_ESM.zip › Source_data/fig01_supplementary/pn_mr_goldens_and_nearby.pdf]

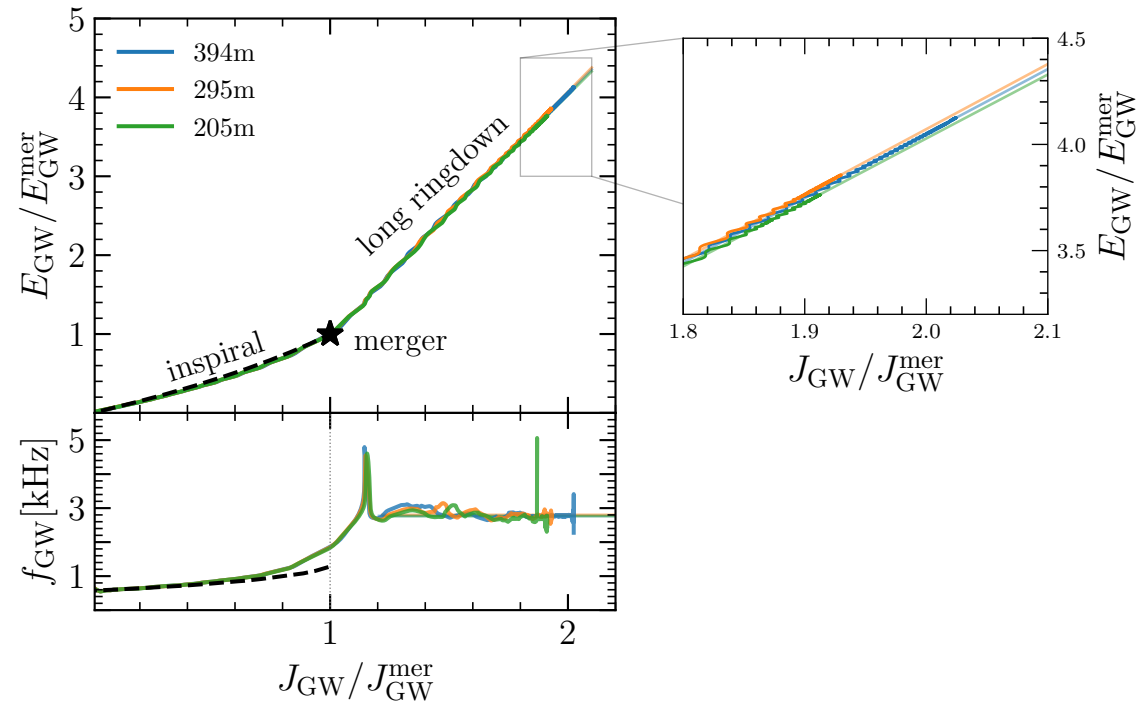

Supplement: Supplementary file 3 — Source Data [file 41467_2025_56500_MOESM3_ESM.zip › Source_data/fig03_supplementary/slopeConvergence.pdf]

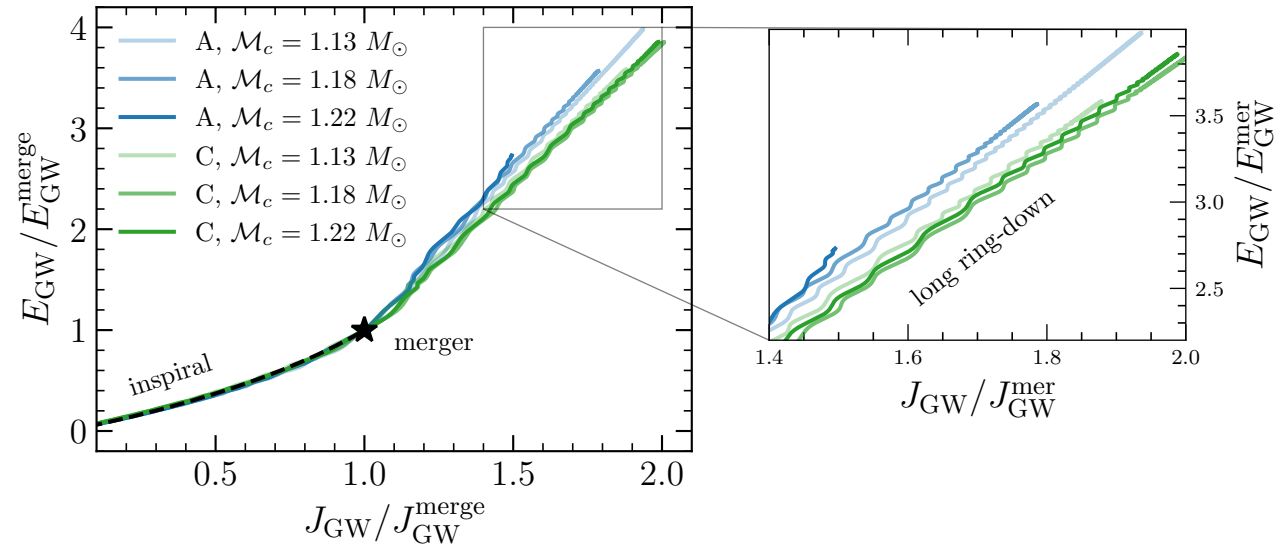

Supplement: Supplementary file 3 — Source Data [file 41467_2025_56500_MOESM3_ESM.zip › Source_data/fig04_supplementary/EJchirp.pdf]

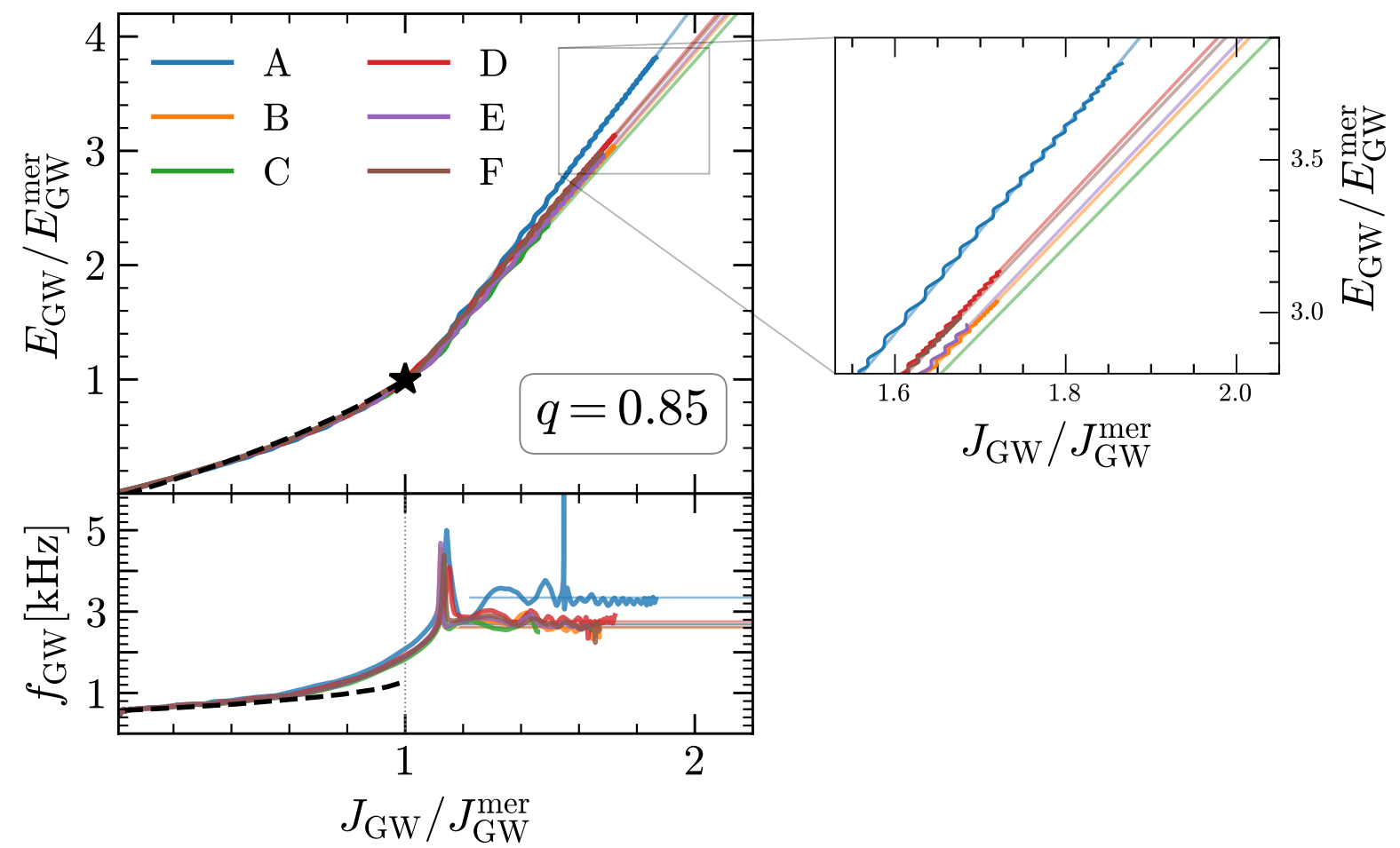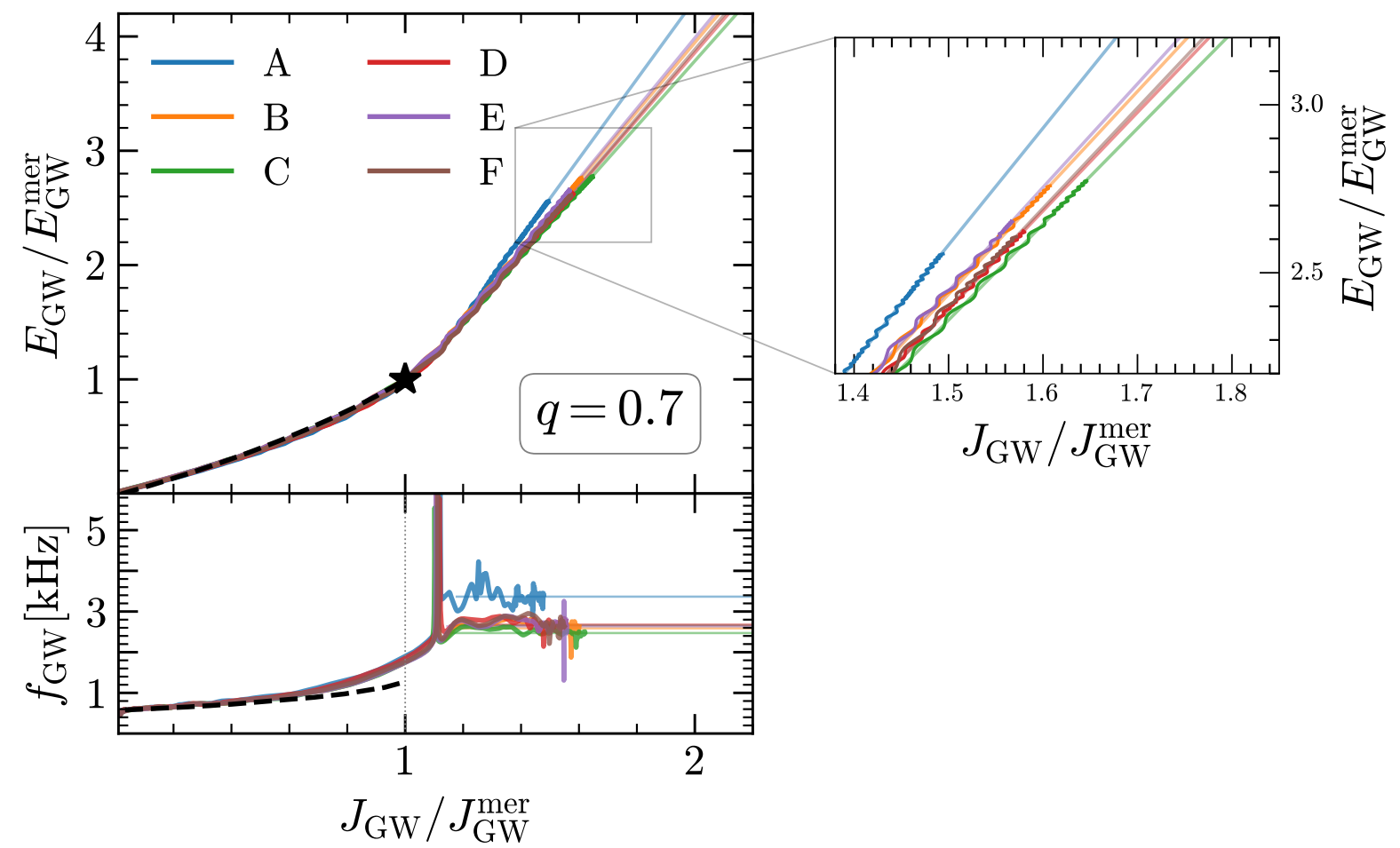

Supplement: Supplementary file 3 — Source Data [file 41467_2025_56500_MOESM3_ESM.zip › Source_data/fig05_supplementary/fig10.pdf]

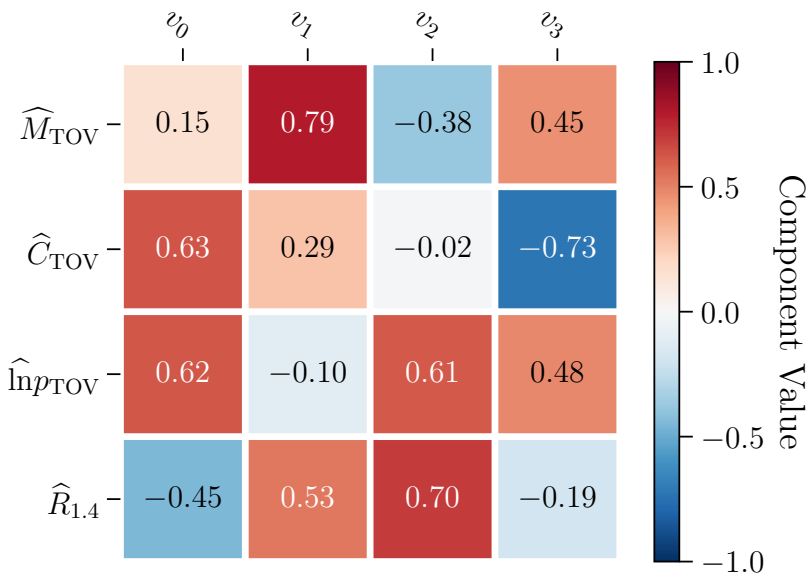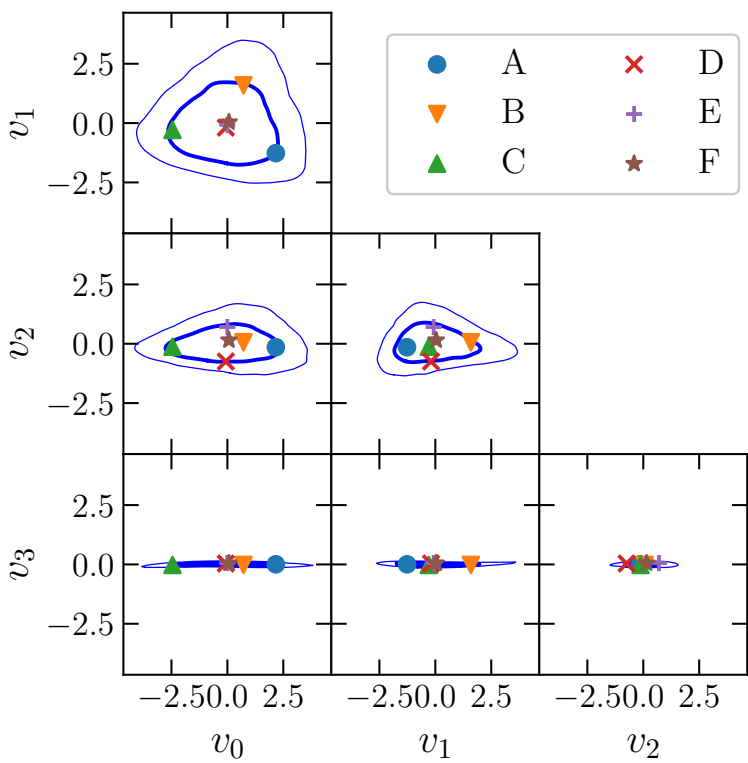

Supplement: Supplementary file 3 — Source Data [file 41467_2025_56500_MOESM3_ESM.zip › Source_data/fig05_main/fig05.pdf]

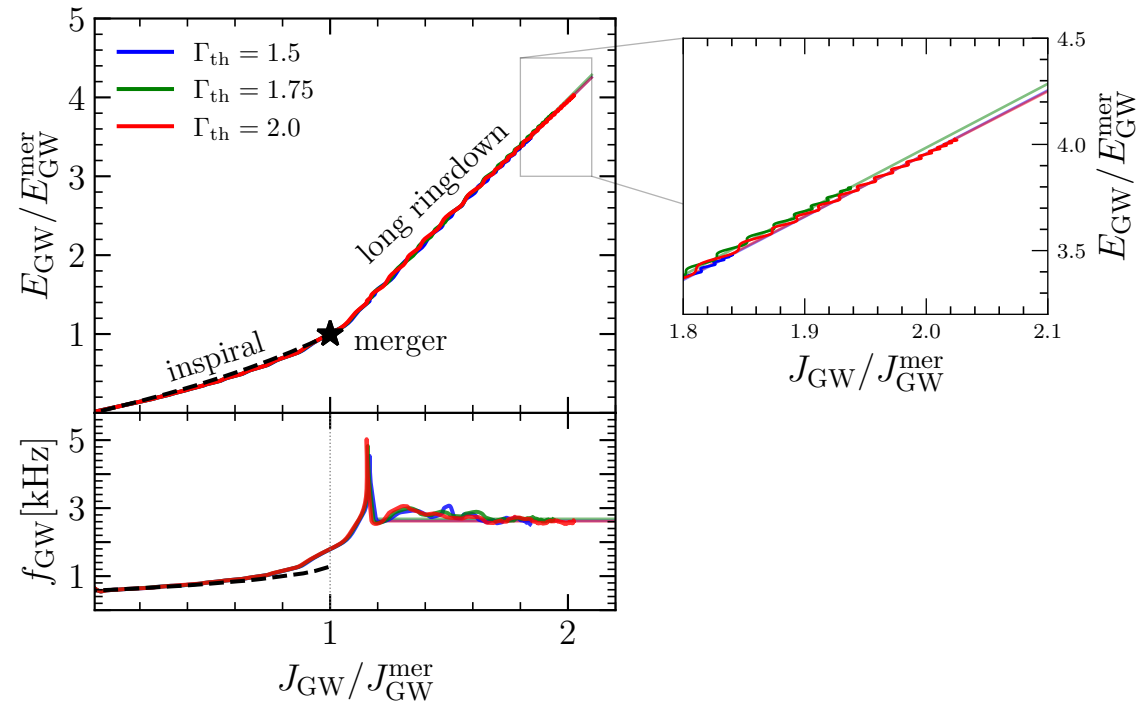

Supplement: Supplementary file 3 — Source Data [file 41467_2025_56500_MOESM3_ESM.zip › Source_data/fig06_supplementary/EJGamma.pdf]

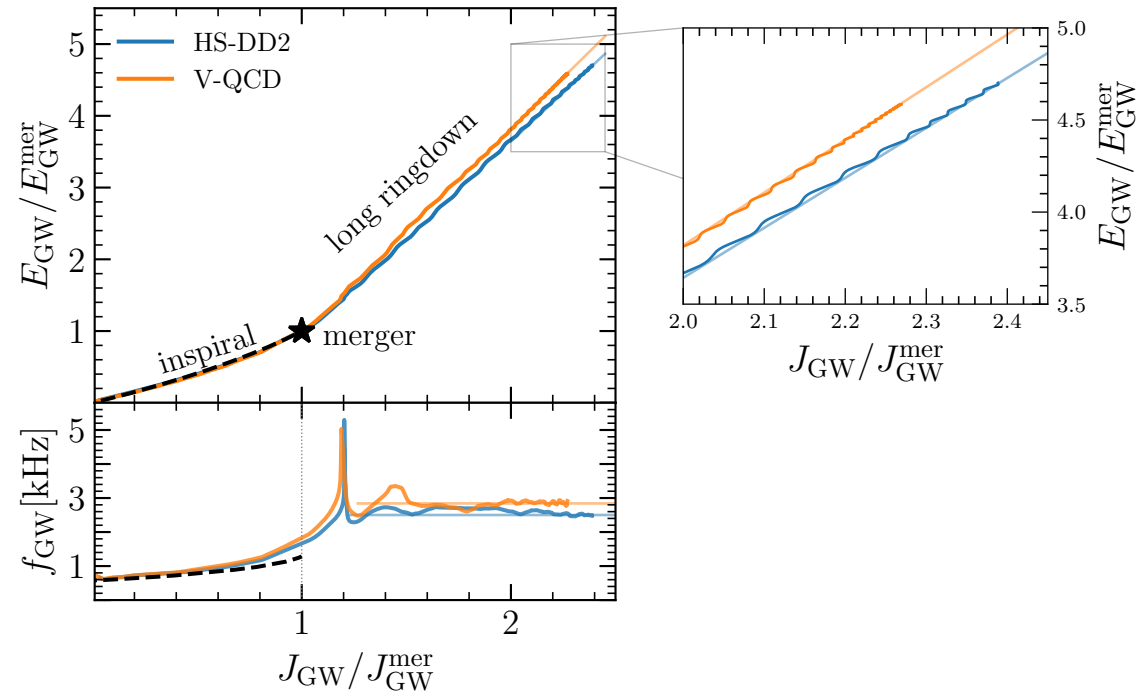

Supplement: Supplementary file 3 — Source Data [file 41467_2025_56500_MOESM3_ESM.zip › Source_data/fig07_supplementary/EJ_and_fmerg_dd2VQCD.pdf]
